# Diffusion MRI harmonization enables joint-analysis of multicentre data of patients with cerebral small vessel disease

**DOI:** 10.1016/j.nicl.2021.102886

**Published:** 2021-11-18

**Authors:** Bruno M. de Brito Robalo, Geert Jan Biessels, Christopher Chen, Anna Dewenter, Marco Duering, Saima Hilal, Huiberdina L. Koek, Anna Kopczak, Bonnie Yin Ka Lam, Alexander Leemans, Vincent Mok, Laurien P. Onkenhout, Hilde van den Brink, Alberto de Luca

**Affiliations:** a Department of Neurology and Neurosurgery, UMC Utrecht Brain Center, University Medical Center Utrecht, Utrecht, The Netherlands; b Image Sciences Institute, University Medical Center Utrecht, Utrecht University, Utrecht, The Netherlands; c Memory, Aging and Cognition Center, Department of Pharmacology, National University of Singapore, Singapore; d Institute for Stroke and Dementia Research (ISD), University Hospital, LMU Munich, Germany; e Medical Image Analysis Center (MIAC AG) and qbig, Department of Biomedical Engineering, University of Basel, Basel, Switzerland; f Department of Geriatric Medicine, University Medical Center Utrecht, Utrecht, The Netherlands; g Division of Neurology, Department of Medicine and Therapeutics, Gerald Choa Neuroscience Centre, Faculty of Medicine, Prince of Wales Hospital, The Chinese University of Hong Kong, Shatin, Hong Kong Special Administrative Region

**Keywords:** Diffusion MRI, Harmonization, Cerebral small vessel disease, Multicentre, White matter hyperintensities

## Abstract

**Objectives:**

Acquisition-related differences in diffusion magnetic resonance imaging (dMRI) hamper pooling of multicentre data to achieve large sample sizes. A promising solution is to harmonize the raw diffusion signal using rotation invariant spherical harmonic (RISH) features, but this has not been tested in elderly subjects. Here we aimed to establish if RISH harmonization effectively removes acquisition-related differences in multicentre dMRI of elderly subjects with cerebral small vessel disease (SVD), while preserving sensitivity to disease effects.

**Methods:**

Five cohorts of patients with SVD (N = 397) and elderly controls (N = 175) with 3 Tesla MRI on different systems were included. First, to establish effectiveness of harmonization, the RISH method was trained with data of 13 to 15 age and sex-matched controls from each site. Fractional anisotropy (FA) and mean diffusivity (MD) were compared in matched controls between sites using tract-based spatial statistics (TBSS) and voxel-wise analysis, before and after harmonization. Second, to assess sensitivity to disease effects, we examined whether the contrast (effect sizes of FA, MD and peak width of skeletonized MD - PSMD) between patients and controls within each site remained unaffected by harmonization. Finally, we evaluated the association between white matter hyperintensity (WMH) burden, FA, MD and PSMD using linear regression analyses both within individual cohorts as well as with pooled scans from multiple sites, before and after harmonization.

**Results:**

Before harmonization, significant differences in FA and MD were observed between matched controls of different sites (p < 0.05). After harmonization these site-differences were removed. Within each site, RISH harmonization did not alter the effect sizes of FA, MD and PSMD between patients and controls (relative change in Cohen’s d = 4 %) nor the strength of association with WMH volume (relative change in R^2^ = 2.8 %). After harmonization, patient data of all sites could be aggregated in a single analysis to infer the association between WMH volume and FA (R^2^ = 0.62), MD (R^2^ = 0.64), and PSMD (R^2^ = 0.60).

**Conclusions:**

We showed that RISH harmonization effectively removes acquisition-related differences in dMRI of elderly subjects while preserving sensitivity to SVD-related effects. This study provides proof of concept for future multicentre SVD studies with pooled datasets.

## Highlights

• RISH harmonization is applicable to multicentre dMRI of elderly subjects with SVD.
• Harmonization removes site-differences in dMRI metrics in matched controls.
• Associations between dMRI metrics and SVD markers in patients are preserved.
• Harmonized scans can be pooled to into a single large dataset to increased power.

## 1 Introduction

Combining data from multicentre studies is becoming increasingly important in neuroimaging, with the aim to increase statistical power and provide outcomes that are more generalizable than those obtained at single-centre level (Lorca-Puls et al., 2018, Van Horn and Toga, 2014). However, joint analysis of multicentre magnetic resonance imaging (MRI) can be challenging if inter-site variability due to acquisition-related inconsistencies is not taken into account (Vollmar et al., 2010).

Inter-site variability is particularly problematic in diffusion MRI (dMRI) and can be caused by a range of factors, including scanner hardware (e.g., scanner manufacturer, magnetic field strength, gradient strength, field inhomogeneities), software, acquisition parameters (e.g., voxel size, number of gradient directions, echo time) (Helmer et al., 2016). All these factors may affect the measured diffusion signal intensity and metrics derived from the data. In prospective multicentre studies, this variability can be controlled using standardized acquisitions and scanners from the same manufacturer (Konieczny et al., 2020). However, when retrospectively combining data form different cohorts, differences in acquisition can be substantial. Even with phantoms, dMRI metrics have shown more than 7% variability across sites (Palacios et al., 2017, Teipel et al., 2011, Timmermans et al., 2019). In the human brain, this variability is even more pronounced and non-uniform across tissues with acquisition-related differences reaching the same order of magnitude as case-control differences (e.g., traumatic brain injury vs. controls, Kumar et al., 2009). In such scenarios, if multicentre data were naively pooled into a single analysis, true biological effects would likely be masked by acquisition-related differences. It is therefore crucial that multicentre dMRI is harmonized prior to joint-analysis (Tax et al., 2019).

Two main kinds of retrospective dMRI harmonization techniques have been developed to date. The first category operates on each diffusion metric individually (e.g., fractional anisotropy – FA, and mean diffusivity – MD) by using statistical approaches such as *meta*-analysis, or by modelling the difference between sites with covariates during analysis (e.g., ComBat, Fortin et al., 2017). By contrast, the second category of harmonization operates directly on the raw dMRI data rather than on each diffusion metric (Karayumak et al., 2019, Koppers et al., 2019, Mirzaalian et al., 2015). This type of harmonization is more general since the raw diffusion signal is harmonized in a model-independent manner, theoretically allowing any type of subsequent analysis. In this study, we focus specifically on the second type of harmonization with the rotation invariant spherical harmonics (RISH) methods (Mirzaalian et al., 2015).

The core idea of the RISH method is to map the dMRI signal from a ‘target’ site to a ‘reference’ site, using groups of healthy subjects matched for factors such as age, sex, etc. This signal mapping is possible because the dMRI signal intensity can be represented in a spherical harmonics (SH) basis with a given number of parametrization coefficients (Tournier et al., 2004). From this representation, RISH features can be extracted and scaled to harmonize the dMRI signal between two sites (Karayumak et al., 2019). Applications of RISH harmonization have been presented using synthetic data and with data of healthy young subjects, with recent work showing that acquisition-related differences are removed while preserving age- and sex-related effects (Karayumak et al., 2019). Recently, this method has also been applied to harmonize a large dataset of patients with Schizophrenia and investigate changes across the lifespan (Cetin-Karayumak et al., 2020). However, the applicability of the RISH method to older individuals exhibiting brain atrophy or to patients with (diffuse) white matter lesions remain unclear.

In this work, we evaluate the RISH harmonization framework in the context of a retrospective multicentre analysis of individuals with brain lesions due to cerebral small vessel disease (SVD). SVD is a leading cause of cognitive impairment and dementia and it is often investigated with dMRI (Baykara et al., 2016, Lyoubi-Idrissi et al., 2017, Wiegertjes et al., 2019). The patterns of diffusion change in SVD are well documented using the diffusion tensor model, with patients typically exhibiting widespread increase of MD, peak width of skeletonized MD (PSMD) and decrease of FA, often related to white matter hyperintensity (WMH) burden (Tuladhar et al., 2015, Van Norden et al., 2012). Hence, this patient population is well-suited to investigate the efficacy of harmonization methods. Using scans from five SVD cohorts acquired on different systems (Philips Healthcare, NL, and Siemens Healthineers, DE) and with different protocols, we aimed to establish if application of the RISH method removes acquisition-related differences in dMRI of elderly subjects, while preserving the sensitivity to disease effects in SVD. Finally, we show proof of concept of how multicentre harmonized data can be pooled to perform robust inference of the relation between WMH burden and dMRI metrics.

## 2 Methods

### 2.1 Datasets and inclusion criteria

For this retrospective analysis, we obtained scans from five cohorts including healthy elderly subjects and patients with SVD. These cohorts differed in study design and inclusion criteria (described below), comprising four samples with sporadic SVD and one sample with genetically defined SVD (Cerebral Autosomal Dominant Arteriopathy with Subcortical Infarcts and Leukoencephalopathy, CADASIL). For the present study, we used a harmonized definition for patients and controls. Patients with sporadic SVD had symptomatic SVD defined as a) history of stroke, with a corresponding small subcortical infarct visible on MRI or b) cognitive complaints and presence of WMH burden on MRI (Fazekas score ≥ 2, Fazekas et al., 1987). The presence of CADASIL was confirmed by molecular genetic testing of the *NOTCH3* gene or ultrastructural analysis of a skin biopsy (detection of pathognomonic granular osmiophilic material, Wollenweber et al., 2015). Patients were excluded if they had other major neurological or psychiatric conditions (e.g., multiple sclerosis, epilepsy, Parkinson’s disease). Controls had no history of stroke or cognitive complaints for which they sought medical advice, and their MRI did not show signs of lacunes or WMH with Fazekas score ≥ 2. All subjects had a structural MRI (T1-weighted) and a dMRI scan. Characteristics of the study samples included in this study (397 patients and 175 controls) are provided in Tables 1. All studies included in this analysis were approved by the ethics committees of the respective institutions and all participants provided written informed consent.

**Table 1:** Demographics and imaging parameters of the study samples.

	Utrecht 1		Hong Kong		Munich		Utrecht 2		Singapore	
	Controls (N = 53)	Patients (N = 171)	Controls (N = 20)	Patients (N = 20)	Controls (N = 34)	Patients (N = 72)	Controls (N = 18)	Patients (N = 34)	Controls (N = 50)	Patients (N = 100)
**Demographics**										
Age, years	71.0 ± 4.8	74.9 ± 9.0	69.2 ± 3.4	74.1 ± 3.3	71.1 ± 4.4	53.4 ± 6.3	62.4 ± 6.9	66.9 ± 8.9	66.6 ± 4.8	72.6 ± 6.9
Male sex (%)	31 (58)	99 (59)	10 (50)	10 (50)	17 (49)	23 (32)	10 (55)	22 (65)	31 (52)	35 (35)
**Cognitive testing**										
MMSE	28 [28–30]	26 [24, 28]	–	–	30 [29, 30]	30 [27,30]	29 [28,30]	29 [27,30]	28 [27,29]	22 [18,25]
MoCA	–	–	25 [23, 27]	19 [14, 20]	–	–	–	–	27 [26,28]	11 [18,21]
**MRI markers**										
WMH volume (% ICV)	0.3 [0.1, 0.6]	1.2 [0.5, 2.7]	0.1 [0.05, 0.1]	0.6 [0.3, 1.1]	0.2 [0.1 0.7]	6.2 [3.5, 10]	0.03 [0.02, 0.08]	0.7 [0.3, 1.3]	0.04 [0.02, 0.09]	0.8 [0.08, 1.16]
WMH (Fazekas)	0 [0, 1]	2 [1, 2]	0.5 [0, 1]	2 [2, 3]	0 [0, 1]	3 [2, 3]	1 [1,1]	2 [2,3]	1 [1,1]	2 [1,3]
Lacunes (present)	0 (0)	69 (36)	0(0)	–	0 (0)	49 (6)	0(0)	17 (50)	0 (0)	37 (37)
**Imaging parameters**										
Scanner	3 T Philips Achieva		3 T Philips Achieva		3 T Siemens Verio		3 T Philips Achieva		3 T Siemens Magnetom Trio, Tim	
Software version	R3.1		MR Release 5.1		syngo MR B19		MR Release 5.6.0		syngo MR B19	
Voxel size (mm^2^)	2.5 × 2.5 × 2.5		1 × 1 × 2		2 × 2 × 2		2.5 × 2.5 × 2.5		3.1 × 3.1 × 3.0	
b-value (s/mm^2^)	1200		1000		1000		1200		1150	
# of directions	45		32		30		45		61	

Data presented as mean ± SD, number (percentages) or median [interquartile range]; SVD = small vessel disease; CADASIL = cerebral autosomal-dominant arteriopathy with subcortical infarcts and leukoencephalopathy; MMSE = Mini-Mental State Examination; MoCA = Montreal Cognitive Assessment; WMH = white matter hyperintensity; ICV = intracranial volume.

#### 2.1.1 Utrecht1

Patients (n = 171) were selected from the Parelsnoer study memory clinic cohort (Aalten et al., 2014). Age-matched controls (n = 53) were recruited from a community-based cohort (Reijmer et al., 2013). All MRI scans fromboth cohorts were acquired on the same 3 Tesla Philips scanner (Achieva, Philips, Best, the Netherlands). T1-weighted scans for both cohorts were acquired with the following parameters: voxel size: 1 × 1 × 1 mm^3^, echo time (TE): 4.5 ms and repetition time (TR): 7.9 ms. dMRI data were obtained with a voxel size: 2.5 × 2.5 × 2.5 mm^3^, TR/TE 6638/73 ms, 45 diffusion gradients directions with a b-value of 1200 s/mm^2^, and 1b = 0 s/mm^2^ averaged 3 times. Fluid-attenuated inversion recovery (FLAIR) images were obtained with TR/TE/inversion time (TI): 11000/125/2800 ms, voxel size: 1 × 1 × 3 mm^3^.

#### 2.1.2 Hong Kong

Patients (n = 20) and controls (n = 20) were selected from a community-based cohort, the Chinese University of Hong Kong–Risk Index for Subclinical brain lesions in Hong Kong (CU-RISK) (Lam et al., 2019). MRI scans were acquired on a 3 Tesla Philips scanner (Achieva, Philips, Best, the Netherlands). T1-weighted images were obtained with TR/TE: 7.49/3.46 ms, voxel size: 0.60 × 1.04 × 1.04 mm^3^ and dMRI had a TR/TE: 8944/60 ms, voxel size: 1 × 1 × 2 mm^3^; 32 diffusion gradient directions with b-value 1000 s/mm^2^ and 1b = 0 s/mm^2^. FLAIR images were acquired with TR/TE/TI: 8000/328.6/2400 ms, voxel size: 0.55 × 0.44 × 0.44 mm^3^.

#### 2.1.3 Munich

Patients (n = 72) with CADASIL and controls (n = 34) were selected from the prospective VASCAMY (Vascular and Amyloid Predictors of Neurodegeneration and Cognitive Decline in Nondemented Subjects) study (Baykara et al., 2016). All MRI scans were acquired on a 3 Tesla Magnetom Verio scanner (Siemens Healthineers, Erlangen, Germany). T1-weighted scans were obtained using TR/TE: 2500/4.73 ms, voxel size: 1 × 1 × 1 mm^3^ and dMRI were acquired with a voxel size: 2 × 2 × 2 mm^3^, TR/TE: 12700/81 ms, 30 diffusion gradient directions with a b-value of 1000 s/mm^2^, and 1b = 0 s/mm^2^. FLAIR images were obtained with TR/TE/TI: 5000/395/1800 ms, voxel size: 1 × 1 × 1 mm^3^.

#### 2.1.4 Utrecht2

A second dataset from the UMC Utrecht consisted of patients (n = 34) and controls (n = 18) from an ongoing prospective observational cohort study Zoom@SVDs (van den Brink et al., 2021). MRI scans were acquired using the same scanner system and acquisition parameters as the Utrecht1 dataset. However, since multiple scanner software and hardware (coil) updates occurred between the two studies, scans from the Zoom@SVDs study are treated as a separate site.

#### 2.1.5 Singapore

Patients (n = 100) and controls (n = 50) were selected from a community-based cohort, the Epidemiology of Dementia In Singapore (EDIS) study (Hilal et al., 2013). All MRI scans were performed on a 3 Tesla Siemens Magnetom Trio Tim scanner (Siemens Healthineers, Erlangen, Germany). T1-weighted scans were obtained with TR/TE: 2300/1.9 ms, voxel size: 1 × 1 × 1 mm^3^ and dMRI were acquired with a TR/TE: 6800/85 ms, voxel size: 3.1 × 3.1 × 3 mm^3^; 61 diffusion gradient directions with b-value 1150 s/mm2 and 7b = 0 s/mm2. FLAIR images were obtained with TR/TE/inversion time (TI): 9000/82/2500 ms, voxel size: 1 × 1 × 3 mm^3^.

### 2.2 MRI data pre-processing

All datasets were pre-processed using ExploreDTI version 4.8.6 (Leemans et al., 2009) and the Functional Magnetic Resonance Imaging of the Brain (FMRIB) software library (FSL, v6.0.1). Images were corrected for signal drift (Vos et al., 2016) , eddy currents, subject motion with rotation of the B-matrix (Leemans and Jones, 2009) , and susceptibility induced distortions (Veraart et al., 2013) . dMRI data were nonlinearly registered to the T1 and resampled to an isotropic resolution of 2 × 2 × 2 mm^3^ and brain masks were generated using brain extraction (BET) tool from FSL. All images were visually inspected to exclude the presence of major artifacts and misregistration. Since the dMRI data were acquired with different b-values in the different cohorts, we adjusted for differences in b-values as part of the harmonization pipeline. We estimated the signal of all dMRI data to a common b-value (b = 1000 s/mm^2^) using a linear scaling of the signal decay (S/S0) in the logarithmic domain (Jensen et al., 2005, Steven et al., 2014). This signal decay has been validated in phantoms and healthy controls and shown to be robust for datasets with closely spaced b-values centred around b = 1000 s/mm^2^ where the diffusion signal is not heavily weighted towards non-Gaussian effects (Magin et al., 2019) . This approach was also utilized by (Karayumak et al., 2019) for the RISH harmonization method. In our case, b-value scaling was applied to Utrecht1, Utrecht2 and Singapore, which had original b-values of 1200 s/mm^2^ and 1150 s/mm^2^.

WMH volumes were segmented from the FLAIR images using an automated pipeline (*coroflo)* and registered the MNI152 template (Kuijf et al., 2019). All volumes were normalized to the percentage of intracranial volume (ICV) of the MNI brain.

### 2.3 Harmonization with RISH features

Harmonization of dMRI with rotation invariant spherical harmonics (RISH) features was first proposed by Mirzaalian et al. 2015, with recent improvements allowing harmonization of scans with different acquisition parameters (Karayumak et al., 2019), which is the case in our study. This type of harmonization is based on the fact that dMRI signal along unique gradient directions can be represented with a basis of spherical harmonics (SH). From this representation, RISH features that describe different aspects of the signal can be calculated. RISH features can be viewed as the total energy at a specific angular frequency (order) in the SH space. The core assumption of this method is that two groups of healthy subjects matched for age, sex, lesion burden, etc., are expected to have similar diffusion profiles on a group level and thus none of the RISH features should be statistically different between sites. Under this assumption, eventual group differences observed in diffusion measurements such as FA and MD are attributed to scanner-related inconsistencies, as previously shown in healthy controls (Ning et al., 2020). To ensure that the average of RISH features captures site properties on a group level and not characteristics of individuals, a minimum number of training controls (15–20) is required from each site (Karayumak et al., 2019). Subsequently, a scaling is determined between the average of RISH features such that scanner-related differences are removed between sites. This mapping is linear in the SH domain, but non-linear in the original diffusion signal domain. We provide a detailed description of RISH harmonization in the supplementary information, and further theory can also be found in the original method papers (Karayumak et al., 2019; Mirzaalian et al., 2015)

**Fig. 1:**
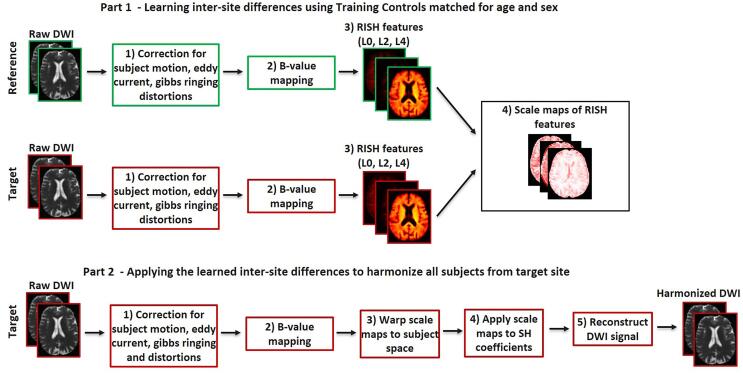
Harmonization steps using RISH features. Part 1) All scans are pre-processed to correct for artefacts, followed by b-value mapping to a common b-value of 1000 s/mm^2^. Voxel-wised scale maps are computed using a set of Training Controls matched for age and sex between the reference and the target site. Part 2) The scale maps are then applied to harmonize the remaining scans of the target site.

In short, RISH harmonization pipeline consists of two parts: 1) learning inter-site differences in the form of scale maps between RISH features of the reference and target site (Fig. 1, part 1); and 2) applying the learned scale maps to harmonize all dMRI datasets of the target site (Fig. 1, part 2). The learning part is performed using age- and sex-matched controls as training data. From the dMRI signal, the RISH features are calculated and registered to a common spatial template generated from training subjects with ANTs (Avants et al., 2010). In the template space, the expected values of RISH features are defined as the sample mean over the number of training subjects and voxel-wise scale maps of RISH features are estimated between the target and reference site. Next, in the application part, the scale maps are warped to the subject space and used to harmonize the SH coefficients of the target site. Finally, the harmonized diffusion signal can be reconstructed.

### 2.4 Experimental design and analysis

#### 2.4.1 Effectiveness of RISH harmonization

Here we assess if acquisition-related differences in diffusion metrics between sites can be removed by RISH harmonization. The first step was to select Training Controls from every cohort that were as similar as possible between sites to minimize sources of variability other than scanner. The Utrecht1 cohort was used as a reference site because the age range of the controls allowed matching with all other sites. This was done on a site-by-site basis, generating four sets of Training Controls with participants from every site matched for age and sex to participants from Utrecht1 (demographics in supplementary Table S1). Tract-based-spatial-statistics (TBSS, Smith et al., 2006) and voxel-based analysis were used to compare the FA and MD between Training Controls of the reference and target sites, before and after harmonization. For the TBSS pipeline, FA and MD were estimated using the diffusion tensor model (*dtfit* from FSL). Next, FA maps were aligned to the MNI152 template and a white matter skeleton representing the centers of major bundles was computed. Subsequently, FA and MD of the skeleton were compared between reference and target sites in a voxel-wise fashion using t-tests with threshold-free cluster enhancement (5000 permutations). This comparison was also extended to the whole brain to ensure that acquisition-related differences in grey matter regions and other structures are also removed.

We also evaluated the generalizability of effectiveness of harmonization beyond the Training Controls. This was done by creating a group of Validation Controls with data from Utrecht1 (n = 15), Munich (n = 15) and Singapore (n = 15), since those sites had a sufficient number of controls outside the Training Controls to generate separate sets of matched groups (demographics in supplementary Table S2). Similar to the analysis with training controls, TBSS and voxel-based analysis were used to compare validation controls between each target site and the reference, before and after harmonization. Furthermore, one-way ANOVA was performed to compare the average FA and MD of the TBSS skeleton across these three sites.

#### 2.4.2 Sensitivity to disease effects in SVD

For this objective we included all patients from all sites (demographics in Table 1). We made a selection of controls that included both training and validation controls, while ensuring that the average age was similar across sites (demographics in supplementary Table S3). We matched controls for age across sites in order to have a common reference to calculate effect sizes against patients. Thus, after harmonization controls are expected to have similar diffusion measures while the contrast with their respective patient groups should not be affected. To assess sensitivity to disease effects, general linear model (GLM) adjusted for age and sex was performed to examine the contrast (effect sizes of FA, MD and PSMD from the TBSS skeleton) between patients and controls within each site, before and after harmonization.

In patients, we also explored the sensitivity to disease effects by relating WMH volume with FA, MD and PSMD, before and after harmonization. Linear regression adjusted for age and sex was performed. Since the WMH volumes was non-normally distributed, a Box-Cox transformation was applied (Box & Cox, 1964). We determined the R^2^ and standardized regression coefficients (β), before and after harmonization.

#### 2.4.3 Proof of concept of data pooling

We evaluated if disease effects were similar if patients were compared to Internal Controls or to a pooled set of matched controls derived from external centres only. GLM adjusted for age and sex was performed to compare patients from Utrecht1 and from Munich versus External Controls pooled from other sites. We compared effect sizes of FA, MD, PSMD obtained with External Controls to the original effect sizes with Internal Controls.

Finally, we demonstrated proof of concept of data pooling by relating WMH volume with FA, MD and PSDM on pooled data of patients from multiple sites, and compared the fit of the curve before and after harmonization. Similar to the analysis within sites, linear regression was performed.

## 3 Results

### 3.1 Effectiveness of RISH harmonization

**Fig. 2:**
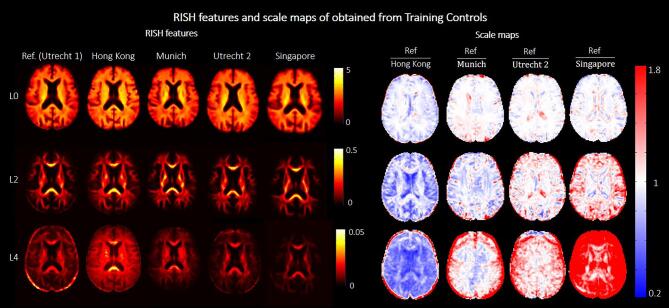
Left (top to bottom): RISH features of order 0, 2 and 4 calculated with Training Controls of each site. Different columns correspond to the different sites. Right (top to bottom): Scale maps for each RISH feature obtained by scaling each target site to the reference. Different columns correspond to different target sites. The red-blue colormap indicates the scaling factor between the two sites. (For interpretation of the references to color in this figure legend, the reader is referred to the web version of this article.)

Fig. 2 shows RISH features of order 0, 2 and 4 obtained from the Training Controls and corresponding scale maps, before harmonization. We observed widespread differences in RISH features between the reference and target sites that were dependent on the tissue type. Small differences were observed in regions with prevalently single fiber populations (e.g., corpus callosum) for all orders of RISH features, whereas more peripheral white matter and grey matter regions showed bigger differences across sites (see scale maps). Furthermore, data from Hong Kong, Munich and Singapore sites showed larger differences from the reference (Utrecht1) than Utrecht2.

**Fig. 3:**
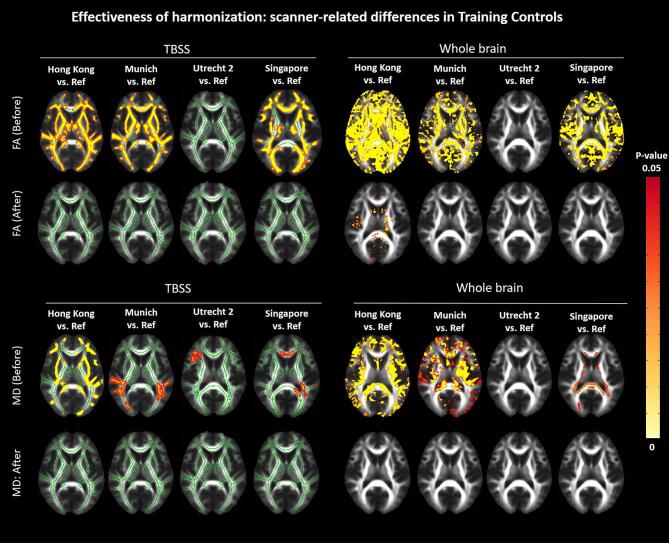
Results of the TBSS (left) and whole brain voxel-wise analysis (right) comparing FA (top) and MD (bottom) between Training Controls of each target site and the reference, before and after harmonization. The yellow–red colormap shows voxels where statistical differences were observed after multiple comparison corrections (p-value < 0.05). Corresponding maps of effect sizes are shown in Supplementary material (Part 3, Fig. S1). (For interpretation of the references to color in this figure legend, the reader is referred to the web version of this article.)

Before harmonization, significant differences in FA and MD were found between the reference and each target site across the entire brain (Fig. 3), especially for Hong Kong Munich and Singapore (p < 0.05). After harmonization, all significant differences in the white matter skeleton between the target sites and the reference were removed. When analysing the whole brain, FA differences were still seen for the Hong Kong site (p < 0.05), mainly in subcortical grey matter and near tissue interfaces with cerebrospinal fluid, probably due to misregistration (Fig. 3, top right panel). A map of effect sizes further clarifies that differences (positive or negative Cohen’s d) are removed after harmonization (i.e., effect sizes become closer to zero, Supplementary Figure S1).

**Fig. 4:**
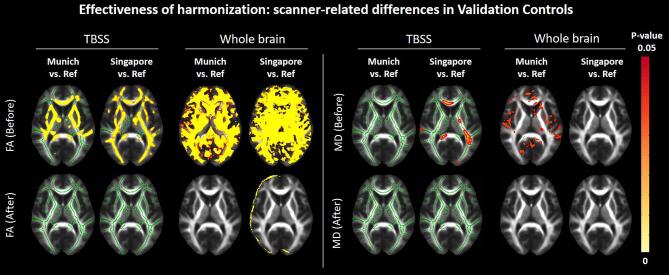
Results of the TBSS and whole brain voxel-wise analysis comparing FA (left) and MD (right) between Validation Controls of each target site and the reference, before and after harmonization (demographics in supplementary Table S2). The yellow–red colormap shows voxels where statistical differences were observed after multiple comparison corrections (p-value < 0.05). (For interpretation of the references to color in this figure legend, the reader is referred to the web version of this article.)

**Fig. 5:**
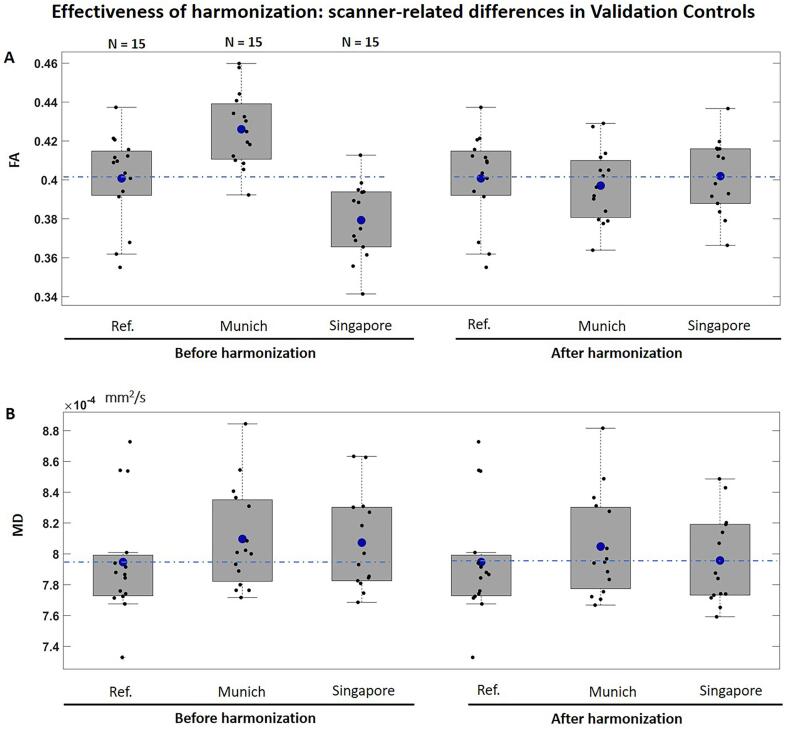
Boxplots of average FA (panel A) and MD (panel B) of the white matte skeleton compared between Validation Controls, before and after harmonization (demographics in supplementary Table S2). The dashed blue line represents the mean of the reference site. The blue marker within each boxplot indicates the mean of the corresponding group, which were compared between sites using a one-way ANOVA. Top: FA before harmonization, F (1,43) = 18.2, p < 0.001; FA after harmonization, F (3,43) = 0.2, p = 0.8. Bottom: MD before harmonization: F (3,43) = 0.83, p = 0.06; MD after harmonization: F (3,43) = 2.6, p = 0.4. (For interpretation of the references to color in this figure legend, the reader is referred to the web version of this article.)

Regarding Validation Controls (Fig. 4), voxel-wise differences in FA and MD in the white matter skeleton and across the whole brain were removed after harmonization, with exception of minor differences at tissue interfaces, probably due to misregistration. When comparing the average FA and MD of the skeleton, significant differences in FA were found across sites before harmonization (F (1,43) = 18.2, p < 0.001, Fig. 5A). All differences in FA were removed after harmonization. The average MD of the skeleton did not differ across sites before or after harmonization (Fig. 5B).

### 3.2 Sensitivity to disease effects in SVD

**Fig. 6:**
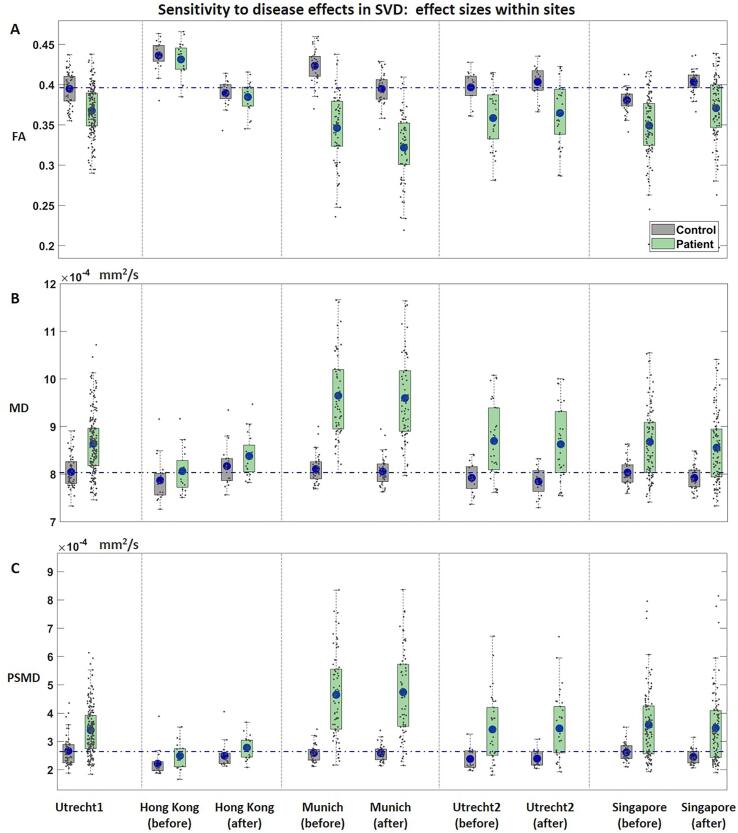
Average FA (panel A), MD (panel B) and PSMD (panel C) of the white matter skeleton compared between patients (green) and controls (gray) within each site (demographics for selected controls in supplementary Table S3). Results are displayed for the reference site and for each target site before and after harmonization. Corresponding p-values and effect sizes are displayed in Table 2. The dashed line indicates the mean value of controls of the reference site. (For interpretation of the references to color in this figure legend, the reader is referred to the web version of this article.)

Fig. 6 depicts differences in dMRI metrics between patients and controls within each site, with quantitative values shown in Table 2. Before harmonization, patients had a significantly lower FA (Fig. 6A), higher MD (Fig. 6B) and higher PSMD (Fig. 6C) than controls in all sites except Hong Kong: FA (d = -0.96 to −2.07, p < 0.001); MD (d = 1.02 to 1.99, p < 0.001); PSMD (d = 0.93 to 1.71, p < 0.001). After harmonization, all effect sizes were preserved, regardless of if they were small (0.2), medium (0.5) or large (0.8). On average, the relative change in effect size from pre- to post-harmonization was 3.9 % (Table 2). Voxel-wise analysis of one of the target sizes (Munich) shows that regional differences between patients and controls are preserved after harmonization (Supplementary Figure S2).

**Table 2:** Effect sizes of FA, MD and PSMD between patients and controls within sites, before and after harmonization.

		Utrecht1	Hong Kong before	Hong Kong after	% Change	Munich before	Munich after	% Change	Utrecht 2 before	Utrecht 2 after	% Change	Singapore before	Singapore after	% Change
FA	P-value	1.40 × 10^−11^	0.43	0.39		3.17 × 10^−21^	2.30 × 10^−21^		2.20 × 10^−5^	2.20 × 10^−5^		3.30 × 10^−10^	9.00 × 10^−15^	
	Effect size (Cohen’s d)	^−^0.96	^−^0.25	^−^0.27	8	−2.07	−2.09	1	−1.16	−1.16	0	−1.19	−1.17	2
	CI	(^−^1.29, ^−^0.64)	(^−^0.87, 0.37)	(^−^0.88, 0.35)		(^−^2.57, −1.57)	(^−^2.59, −1.59)		(^−^1.79, ^−^0.51)	(^−^1.79, ^−^0.52)		(^−^1.54, ^−^0.83)	(^−^1.53, ^−^0.81)	
MD	P-value	6.29 × 10^−10^	0.17	0.13		9.57 × 10^−21^	6.29 × 10^−21^		2.84 × 10^−4^	2.62 × 10^−4^		2.40 × 10^−14^	5.80 × 10^−14^	
	Effect size (Cohen’s d)	1.02	0.44	0.48	9	1.99	2	1	1.18	1.19	1	1.14	1.11	3
	CI	(0.63, 1.34)	(^−^0.19, 1.06)	(^−^0.15, 1.11)		(1.49, 2.49)	(1.49, 2.48)		(0.54, 1.82)	(0.55, 1.83)		(0.80, 1.50)	(0.76, 1.50)	
PSMD	P-value	3.10 × 10^−12^	0.06	0.04		3.6 × 10^−17^	5.97 × 10^−18^		3.2 × 10^−6^	9 × 10^−6^		4.10 × 10^−12^	2.40 × 10^−12^	
	Effect size (Cohen’s d)	0.93	0.62	0.69	11	1.71	1.78	4	1.03	1.1	7	^−^0.99	^−^0.99	0
	CI	(0.62, 1.25)	(^−^0.02, 1.25)	(0.05, 1.32)		(1.23, 2.18)	(1.29, 2.25)		(0.39, 1.65)	(0.46, 1.73)		(^−^1.34, ^−^0.64)	(^−^1.35, ^−−^0.65)	

Demographics for selected controls in Supplementary Table S3.

**Fig. 7:**
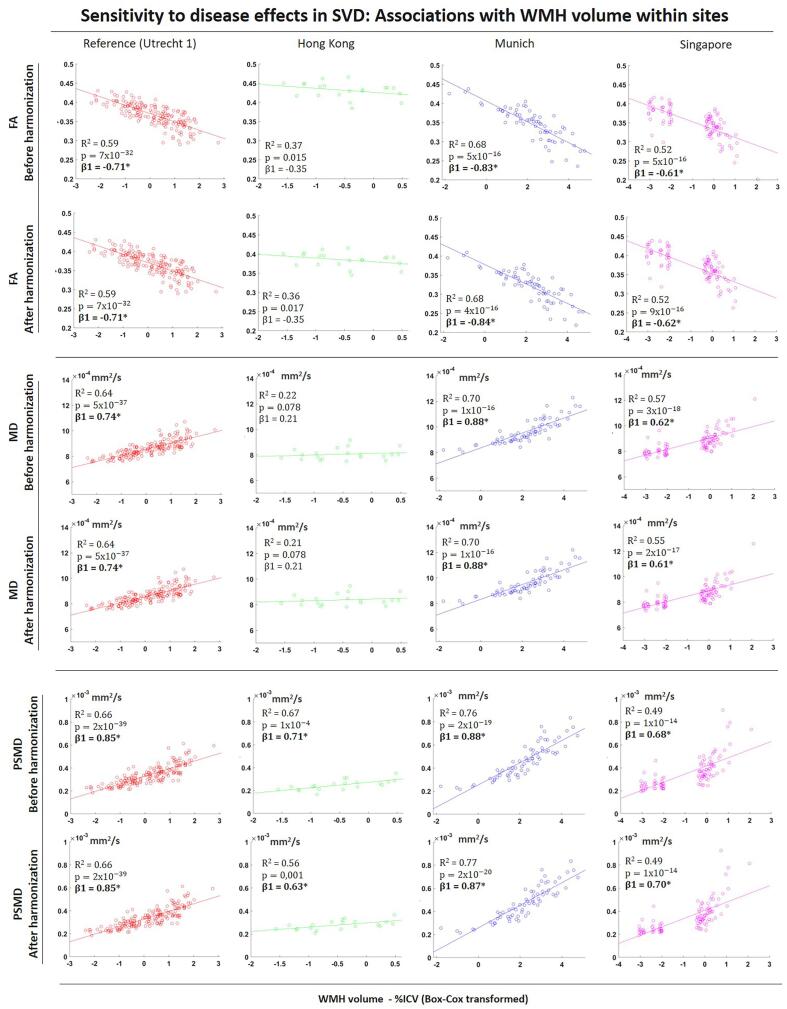
Scatter plots of associations between WMH volume, FA (top), MD (middle) and PSMD (bottom) within sites, before and after harmonization. MD and PSMD values are given in mm^2^/s. *p < 0.05 for β coefficients.

Before harmonization, WMH volume was significantly associated with all dMRI metrics in all sites, FA (R^2^ = 0.37 to 0.68, p < 0.001); MD (R^2^ = 0.57 to 0.70, p < 0.001); PSMD (R^2^ = 0.49 to 0.76; p < 0.001), except Hong Kong where associations MD were not significant (Fig. 7). After harmonization, all associations were preserved regardless of the strength, with R^2^, standardized β coefficients being marginally affected. The relative change in R^2^ after harmonization was 2.8%.

### 3.3 Proof of concept of data pooling

**Fig. 8:**
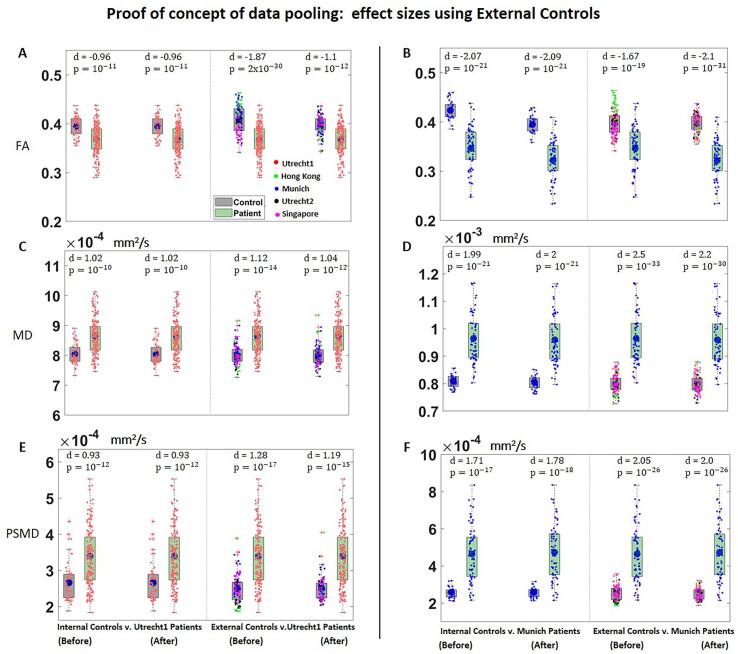
Boxplots of FA (A-B), MD (C-D) and PSMD (E-F) of the white matter skeleton compared between patients and controls. (Left): Comparisons between Utrecht1 patients and Internal Controls and between Utrecht1 patients and External Controls, before and after harmonization. (Right): Comparisons between Munich patients and Internal Controls, and between Munich patients and External Controls, before and after harmonization (demographics for selected controls in supplementary Table S3). Data from each site is color coded as follows: red = Utrecht1; green = Hong Kong; blue = Munich; black = Utrecht2; magenta = Singapore. (For interpretation of the references to color in this figure legend, the reader is referred to the web version of this article.)

When comparing Utrecht1 patients to External Controls before harmonization, differences in FA were close to twice as large as the original effect size obtained with Internal Controls from Utrecht1 (d = -1.87, compared to −0.96, Fig. 8A). After harmonization, the effect size of FA between Utrecht1 patients and External Controls was more comparable to the original effect sizes (Cohen’s d = -1.1, compared to −0.96). Results were similar when we performed the same analysis using patients from Munich (Fig. 8B)): effect sizes between patients and the External Controls were more similar to the original effect size after harmonizing the data (original effect size: d = -2.07; effect size with External Controls before harmonization d = -1.67, after harmonization d = -2.1). For MD and PSMD, the effect sizes between patients and External Controls were similar to the original effects for both Utrecht1 and Munich, even before harmonization (Fig. 8 C-F).

**Fig. 9:**
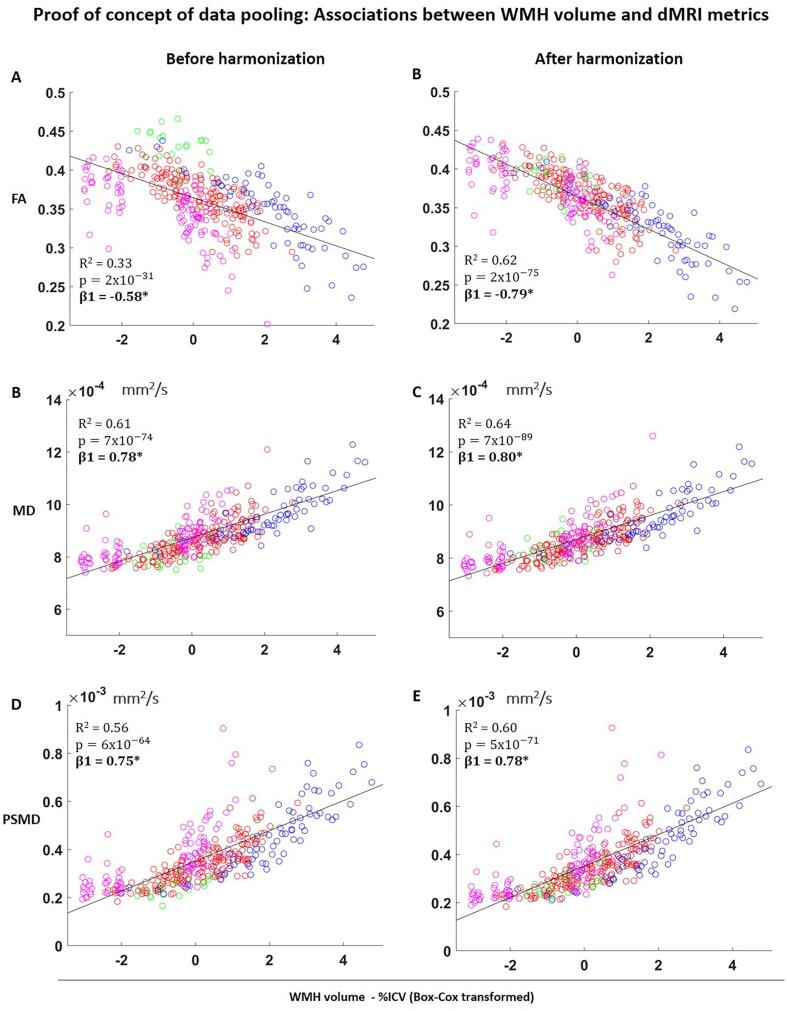
Scatter plots of associations between WMH volume, FA (top), MD (middle) and PSMD (bottom) on the pooled data, before and after harmonization. MD and PSMD values are given in mm^2^/s. *p < 0.05 for β coefficients. Data from each site is color coded as follows: red = Utrecht1; green = Hong Kong; blue = Munich; magenta: Singapore. (For interpretation of the references to color in this figure legend, the reader is referred to the web version of this article.)

Regarding associations between WMH and FA on the pooled data before harmonization, FA values from different sites were clustered in separate clouds (Fig. 9A). This non-harmonized data still described a significant association between WMH volume and FA but with weaker correlations than some individual sites due to the clustering effect (R^2^ = 0.33; p = 2 × 10^-31^). After harmonization, data points were more aligned around the fitted curve, with the measurements behaving as a single center data (Fig. 9B). This resulted in stronger associations between WMH volume and FA (R^2^ = 0.62; p = 2 × 10^-75^). For MD (Fig. 9 C-D) and PSMD (Fig. 9 E-F), the clustering of points was less prominent, but associations with with WMH volume also became stronger after harmonization. Before harmonization, MD (R^2^ = 0.61; p = 7 × 10^-74^); PSMD (R^2^ = 0.56; p = 6 × 10^-64^); after harmonization, (MD: R^2^ = 0.64; p = 7 × 10^-89^); PSMD (R^2^ = 0.60; p = 5 × 10^-71^).

## 4 Discussion

We investigated the applicability of RISH harmonization to remove acquisition-related differences in multicentre dMRI of elderly subjects with SVD while preserving disease-related effects. Before harmonization, we observed significant differences in FA and MD across sites, which were removed after harmonization, both in the Training Controls and in Validation Controls not involved in the training step. Importantly, effect sizes of FA, MD and PSMD for group differences between patients and controls as well as for associations with WMH volume within each site were preserved after harmonization. The harmonized controls could be effectively considered as a single-site dataset. The pooled data of patients covered a wide range of WMH burden, allowing to demonstrate a strong relation between WMH volume and dMRI metrics.

The RISH method has been previously implemented using scans of healthy young subjects without apparent brain lesions for the training step (Cetin-Karayumak et al., 2020, Mirzaalian et al., 2015) . Here, we have evaluated whether peculiar characteristics of the elderly brain, such as presence of lesions as WMH brain atrophy and larger ventricles, which are present to some extent even in control subjects, could affect the computation of the scale maps of RISH features. In our study, the selection criteria for controls was based on low burden of SVD. Thus, the training controls were minimally affected by WMH and had relatively similar brain volumes. The largest differences in RISH features were observed in grey matter and peripheral white matter areas, where partial volume effects might play a role on the diffusion profile (Vos et al., 2011, Vos et al., 2012). After harmonization differences in FA and MD between the target sites and the reference were removed across the brain, except for Hong Kong where minor differences in MD still persisted in deep grey matter structures and at tissue interfaces with cerebrospinal fluid. This is likely due to residual inaccuracies in image registration or differences in WMH burden and brain volumes in Training controls, given these were not explicitly matched for these markers (Supplementary Figure S7). Accordingly, we suggest that when dealing with data of elderly subjects it might be beneficial to match Training Controls not only for age and sex (Hsu et al., 2008) , but also in terms of WMH lesion distribution, brain volumes or other demographics that contribute for variation in diffusion (e.g., handedness, race, etc., Büchel et al., 2004), although this might be challenging to achieve in practice in most studies. This is particularly important when the inclusion criteria for controls are not based on MRI markers of SVD but rather on variables such as being cognitively healthy. Another important consideration for studies implementing the RISH method is that imaging artefacts specific of one site or few subjects (e.g., ghosting, incomplete fat saturation) might be learned as part of the harmonization features, and propagate into the harmonized dataset. In our study, an example of these artefacts can be seen as rings due to incomplete fat suppression on the L4 scale maps shown in Fig. 2. Nevertheless, their impact on the harmonized data was deemed minimal and it did not significantly affect any subsequent result. The RISH method does not assume that two groups of healthy older subjects are completely identical, but as shown by our work, if groups are matched for major factors, differences in RISH features can be learned on a group-level, without major influence of individual properties. This is further supported by our results on the generalizability of RISH harmonization with Validation Controls. We demonstrated not only that dMRI metrics of subjects not involved in the training step are harmonized, but also the transitivity of harmonization, e.g., that the independent harmonization of two target sites to the reference also implies harmonization between target sites.

Next, we demonstrated that RISH harmonization does not affect the sensitivity of dMRI to effects of SVD. Well-known differences in FA, MD and PSDM between patients and controls observed within each site before harmonization were preserved (Baykara et al., 2016, Wiegertjes et al., 2019). Effect sizes were unaffected after harmonization (relative change = 3.9 %.), regardless of whether the magnitude was small, medium or large. We believe such small change in effect size can be deemed negligible, and is likely caused by registration and interpolation inaccuracies (Karayumak et al., 2019). We also repeated the same analysis with the statistical harmonization method ComBat (Fortin et al., 2017) for comparison. RISH harmonization outperformed ComBat in this dataset, which was not able to preserve effect sizes within all sites (see supplementary Information, Part 6). Recent work has shown that application of the RISH method does not alter the relation between dMRI metrics and biological effects such as age-related changes (Karayumak et al., 2019; 2020). Here, we extended such finding by showing that RISH harmonization also preserved the relation between WMH volume and dMRI metrics, a well-established relation in this kind of patients (Van Leijsen et al., 2017), regardless of the strength or the sample size available within each site to test such associations.

To date, most studies of SVD with dMRI have been single-site based, and the inclusion of cohorts from other sites, which can differ substantially in terms of acquisition, has been limited to external validation only. Our results with External Controls indeed show that multicentre data without standardized acquisition across centres cannot be simultaneously analysed, as their integration before harmonization would result in biased effect sizes when comparing patients and controls. Effect sizes obtained with External Controls before harmonization were biased up to 1 standard deviation, which is on the same order of magnitude as typical differences between patients and controls. Conversely, after harmonization External Controls behaved as single-site dataset that could be used as reference for patients from all sites with minimal bias in effect sizes. An important implication of this result is that harmonization can potentially address data obsolescence. Diffusion scans are routinely acquired at single-site level for the purpose of testing specific hypotheses, and discarded afterwards as hardware updates are implemented or the acquisition protocols are adjusted. Being able to account for such differences might allow to include previously acquired data across multiple studies, thus valorising previous investments and reducing the burden of scanning new controls in prospective studies. Another potential benefit of harmonization is in longitudinal studies where upgrades in scanner systems complicate the comparison of data at different time points (Takao et al., 2012). Still, for prospective multi-center studies and especially clinical trials, which typically lack a healthy control group needed for post-hoc data harmonization, standardization of the acquisition should still have a high priority.

After establishing that RISH harmonization allows to integrate data from different cohorts, we demonstrated associations between WMH volume and dMRI metrics on the pooled dataset of patients, which resulted into improved statistical power due to the larger sample size. Since the cohorts included in this study had different disease burdens, the pooled data covered a larger spectrum of WMH volumes, allowing to test associations with more confidence than what the individual sites would allow. Since WMH volume already has strong correlations with dMRI metrics (Bendlin et al., 2010), even the non-harmonized pooled data resulted in significant associations. However, R^2^ were lower than some individual sites, showcasing again the risk of performing inferences using non-harmonized data. After harmonization, data points of different sites behaved as a single-center data and more aligned with the fitted curve, resulting in and increase of R^2^. The impact of harmonization is likely to be even more important in the study of other correlates of SVD with more subtle effect sizes than WMH (e.g., relation between dMRI metrics and cognition, Du et al., 2020) or when assessing disease progression over time (van Leijsen et al., 2019).

## 5 Limitations and future directions

Despite its advantages, RISH harmonization does not come without limitations. To minimize differences in diffusion weighting across sites, we used a linear scaling to map the diffusion signal to a b-value of 1000 s^2^/mm, which is only applicable to a limited range of b-values. For prospective multicentre studies, it is crucial to minimize differences in acquisition parameters such as b-values, but for retrospective studies with already acquired data, harmonization of scans with largely different b-values needs further investigation. As clinical protocols continuously improve and more complex sequences are implemented (e.g., multi-shell data), future work should also investigate whether RISH harmonization is suitable for such advanced dMRI applications in SVD (De Luca et al., 2018, Konieczny et al., 2020, Rydhög et al., 2017). Moreover, since we focused our analyses on the dMRI metrics most commonly associated with SVD (FA, MD, PSMD), further analyses are required in order to generalize our conclusions to other metrics obtained from higher level analysis such as fiber tractography (De Luca et al., 2020) and network theory in SVD (Reijmer et al., 2015).

## 6 Conclusions

Despite the limitations, our study is the first to prove the feasibility of RISH harmonization of multicentre dMRI scans in the context of SVD. We showed that harmonizing the raw dMRI signal is effective in removing acquisition-related differences, while preserving the sensitivity to disease effects. This ultimately allowed us to directly pool scans acquired at different sites into a single analysis and increase the power of dMRI inferences. Our work paves the way not only for the validation of dMRI markers of SVD in large scale multicentre studies, but also for studies that aim to answer new research questions where statistical power is critical, such as untangling underlying aetiologies in SVD populations (e.g., free-water imaging, Finsterwalder et al., 2020). When translated to even larger scales, harmonization could significantly improve the sensitivity and specificity for studies attempting to identify tract specific damage and network connections related to cognitive dysfunction (Konieczny et al., 2020).

## 7 Data availability statement

The data used in this study is available upon contact and agreement with the respective investigators of each cohort. The harmonization method used in this work was originally developed by Mirzaalian et al., 2015 and the code is publicly available at https://github.com/pnlbwh/dMRIharmonization.

## Declaration of Competing Interest

The authors declare that they have no known competing financial interests or personal relationships that could have appeared to influence the work reported in this paper.
