# Supplementary material for: Diffusion MRI harmonization enables joint-analysis of multicentre data of patients with cerebral small vessel disease
Source: Neuroimage Clin. 2021 Nov 18;32:102886. doi: 10.1016/j.nicl.2021.102886 (PMC8609094; doi:10.1016/j.nicl.2021.102886)
Supplement: Supplementary data 1 [file mmc1.docx]

# Supplementary Information

# Part 1 - Tables

**Table S1:** Demographic characteristics Training Controls used to learn scanner differences and compute voxel maps of RISH features between the reference and target sites.

|  | Hong Kong vs Reference | | Munich vs Reference | | Utrecht2 vs Reference | | Singapore vs Reference | | Munich vs Reference  (retraining) | |
| --- | --- | --- | --- | --- | --- | --- | --- | --- | --- | --- |
|  | Hong Kong  (N = 15) | Reference  (N = 15) | Munich  (N = 15) | Reference  (N = 15) | Utrecht2  (N = 13) | Reference  (N = 13) | Singapore  (N = 15) | Reference  (N = 15) | Munich  (N = 15) | Reference  (N = 15) |
| Demographics |  |  |  |  |  |  |  |  |  |  |
| Age, years | 69.0 ± 3.2 | 68.7 ± 3.4 | 70.6 ±3.5 | 70.8 ±4.4 | 65.0 ±3.0 | 65.5 ±0.5 | 70.6 ±2.8 | 70.8±4.4 | 69.4 ±5.1 | 69.4 ±4.1 |
| Male sex (%) | 47 | 47 | 53 | 53 | 61 | 53 | 53 | 53 | 47 | 47 |
| Total brain volume (%ICV) | 78.1 ± 9.1 | 74.4 ± 5.8 | 76.9 ± 3.2 | 73.9 ± 3.8 | 81.1 ± 8.2 | 79.4 ± 6.0 | 78.1 ± 9.1 | 74.1 ± 9.4 | 79.0 ± 4.1 | 74.8 ± 5.5 |

Each target site (Hong Kong, Munich, Utrecht2) was matched to the reference site (Utrecht1) for age and sex. In the matched groups, no significant differences were observed for both covariates (p > 0.05). Munich data was training twice using a second selection of Training Controls, to ensure that harmonization is not affected by sampling bias (results in section 2 of the supplementary information). ICV – Intracranial volume.

**Table S2:** Demographic characteristics of Validation Controls.

|  | Reference  (N=15) | Munich  (N=15) | Singapore  (N=15) |
| --- | --- | --- | --- |
| Demographics |  |  |  |
| Age, years | 70.8 ± 3.4 | 70.9 ± 5.7 | 68.6 ± 5.6 |
| Male sex (%) | 67 | 47 | 60 |

For each site, controls that were not involved in the training step were selected as Validation Controls, to test generalizability of harmonization. This was done for Utrecht1, Munich and Singapore, since those sites had a sufficient number of controls to generate separate sets of matched groups.

**Table S3:** Demographic characteristics of controls used to test effect sizes vs. patients.

|  | Utrecht1  (N = 44) | Hong Kong  (N = 20) | Munich  (N = 30) | Singapore  (N = 34) | Utrecht2  (N = 12) |
| --- | --- | --- | --- | --- | --- |
| Demographics |  |  |  |  |  |
| Age, years | 68.8 ± 3.6 | 69.2 ± 3.4 | 69.3 ± 4.3 | 68.3 ± 4.9 | 68.4 ± 3.4 |
| Male sex (%) | 54 | 50 | 40 | 59 | 66 |

This selection of controls includes both Training and Validation Controls. Age-matching of controls across sites was applied to reduce between site differences in controls. We made no attempt to also age-match the patients across sites, as this was not required for the intended analyses.

**Part 2 - Detailed descriptions of RISH harmonization**

### 2.1 Diffusion MRI signal and RISH features

The use of rotation invariant spherical harmonics (RISH) for harmonization was first proposed by Mirzaalian et al. (2015), with recent improvements allowing harmonization of datasets with different acquisition parameters (Karayumak et al, 2019), which is the case in our study. This harmonization method is based on the fact that dMRI signal *S* = [*S_1_...S_G_*]*^T^* along unique gradient directions (*G*) can be accurately represented in a basis of spherical harmonics (SH) with coefficients *C_ij_* given by: *S* ≈ ∑∑ *C_ij_ Y_ij_* , where *Y_ij_* are SH basis functions of order *i* and phase *j*. From this representation, several RISH features $\mathcal{F}$ per harmonic order are computed as follows (Mirzaalian et al. 2015):

$$\mathcal{F=}\left\| \left. C_{0} \right\| \right.^{2} \left\| \left. C_{2} \right\| \right.^{2}\ldots\left\| \left. C_{8} \right\| \right.^{2} \mathrm{where}\left\| \left. C_{i} \right\| \right.^{2}= \sum_{j=1}^{2i+1} \left( C_{ij} \right)^{2} (1)$$

RISH features can be viewed as the total energy of the signal in a particular frequency band (order) in the SH space and capture different aspects of the signal. Given two groups of healthy subjects matched for age and sex, they are expected to have similar diffusion profiles and thus the RISH features should not be statistically different on a group level. Therefore, differences found in diffusion measurements are mainly attributed to scanner-related inconsistencies. During the harmonization procedure, the goal is to determine appropriate scaling factors for RISH features between the reference and target sites. This mapping is linear in the SH domain, but non-linear in the original diffusion signal domain.

### 2.2 Mapping voxel-wise RISH features in training controls

We estimated voxel-wise linear mapping between each target site and the reference site using training controls, as depicted in Part 1 of Figure 1. We computed 3 RISH features of order 0, 2 and 4 which capture different microstructural aspects of the signal. We did not calculate higher order RISH features since in our dataset they primarily capture noise. Next, RISH feature maps were registered to create a multi-modal template using ANTs algorithm (Avants et al., 2010). In the template space, the expected values of the voxel-wise RISH features are defined as the sample mean over the number of training subjects:

$$\mathbb{E}_{i}^{s} \approx\sum_{n=1}^{N_{t}} \left[ \left\| C_{i}^{n} \right\|^{2} \right]/N_{s} (2)$$

Where $C_{i}^{n}$ is the RISH feature of order *i* for the n^th^ subject and $N_{s}$ is the number of training subject for site *s*. From here voxel-wise scale maps between RISH features of target site (TAR) and reference site (REF) are computed in the template space using:

$$\phi(REF, TAR)\approx\sqrt{\frac{\mathbb{E}_{i}^{REF}}{\mathbb{E}_{i}^{TAR}+ ɛ}} \left( 3 \right)$$

Where ɛ is a small non zero constant.

### 2.3 Applying scale maps to harmonize all DWIs

The scale maps estimated from the training data were applied to all other scans of the target site. First the scale maps are nonlinearly transformed to the native subject space. This transformation is obtained by registering the RISH features of each subject to the template space and then inverting the transformation. The dMRI signal can now be recalculated by scaling the SH coefficients at each voxel in the subject space with:

$$\hat{C}_{ij} = \phi(REF, TAR)\hat{C}_{ij} \left( 4 \right)$$

Where $\hat{C}_{ij}$ are the scaled SH coefficients. Finally, the harmonized diffusion signal in the SH domain is transformed back to the signal intensity domain as follows:

$$\hat{S} = \sum\sum\hat{C}_{ij} Y_{ij} \left( 5 \right)$$

**Part 3 -Effectiveness of harmonization in training controls: effect size maps**


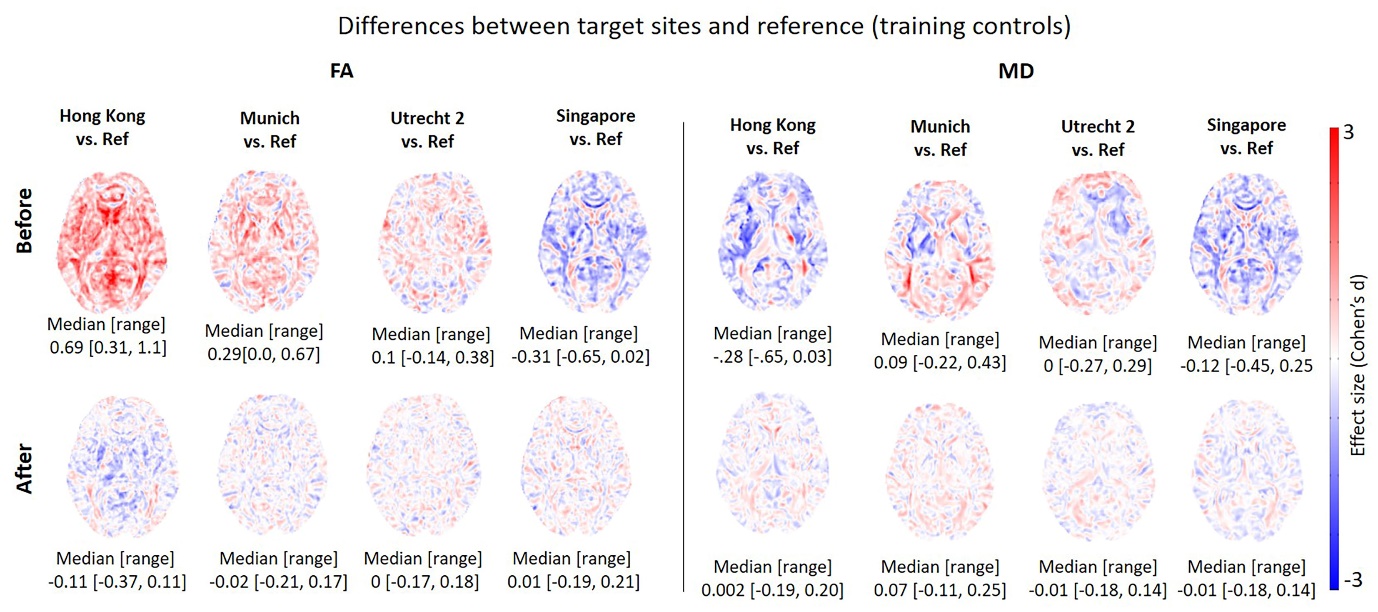


**Figure S1:** Results of whole brain voxel-wise analysis (right) comparing FA (top) and MD (bottom) between Training Controls of each target site and the reference, before and after harmonization. The red-blue colormap shows Cohen’s d effect sizes. These maps support Figure 3, showing that effect sizes become close to zero after harmonization.

**Part 4 - Sensitivity to disease effects: voxel-wise differences between patients and controls**


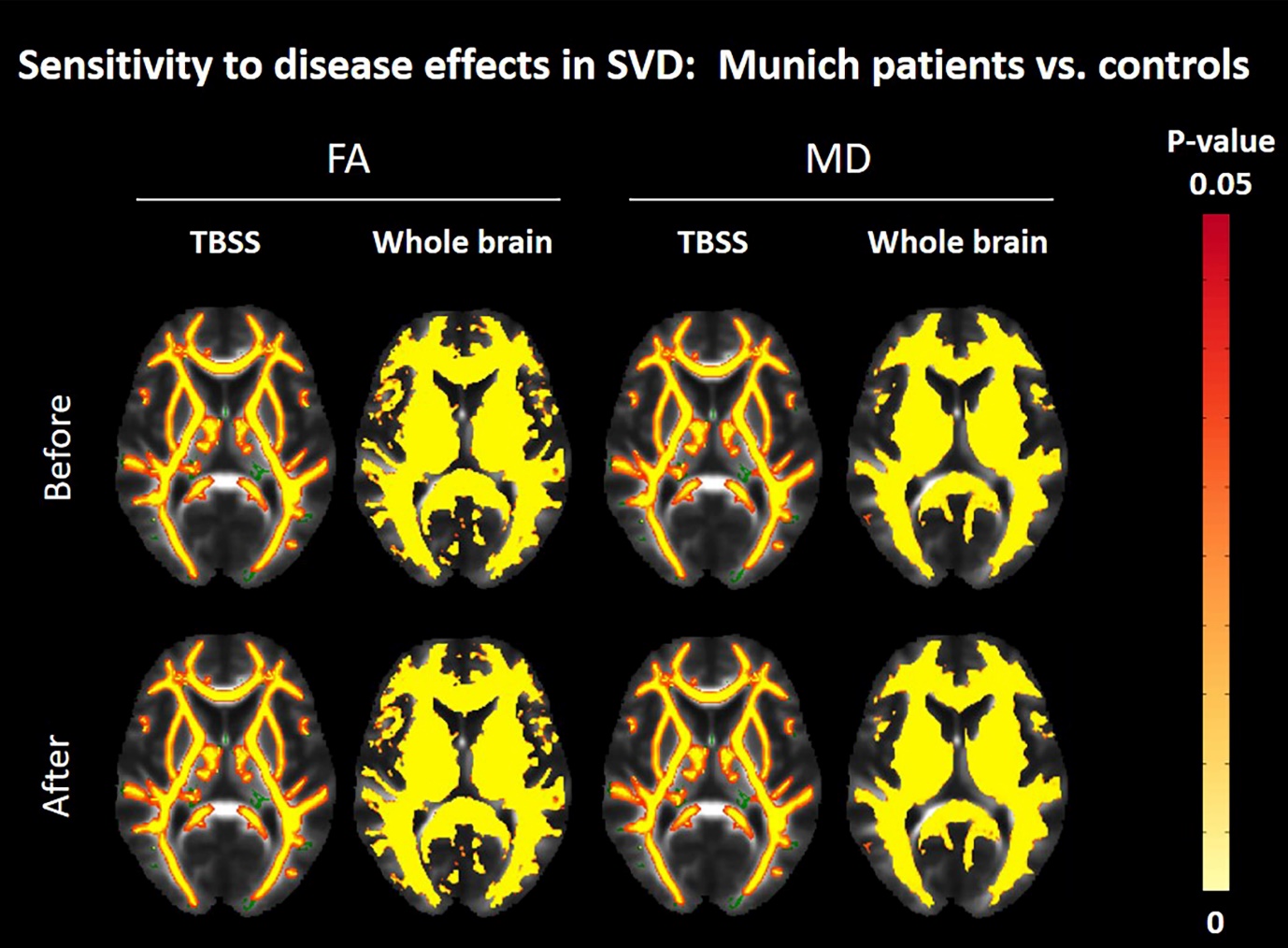


**Figure S2:** Voxel-wise differences in FA and MD between patients and controls (in Munich), before and after harmonization. Colormap shows p < 0.05

**Part 5 - Effectiveness of harmonization using a second sample Training Controls**

In Figure S1 we demonstrate that RISH harmonization is independent of the sample of training subjects used to train the algorithm, as long as these subjects are matched between the reference and the target. Differences between matched controls of Utrecht1 and Munich sites found before harmonization were removed after harmonization (note that the gray boxplots representing the controls fall on the same line after harmonization). Furthermore, within each site, effect sizes of FA, MD, PSMD found before harmonization were unaffected after harmonization. This result indicates that the RISH method is independent of sampling biases of Training Controls.

**
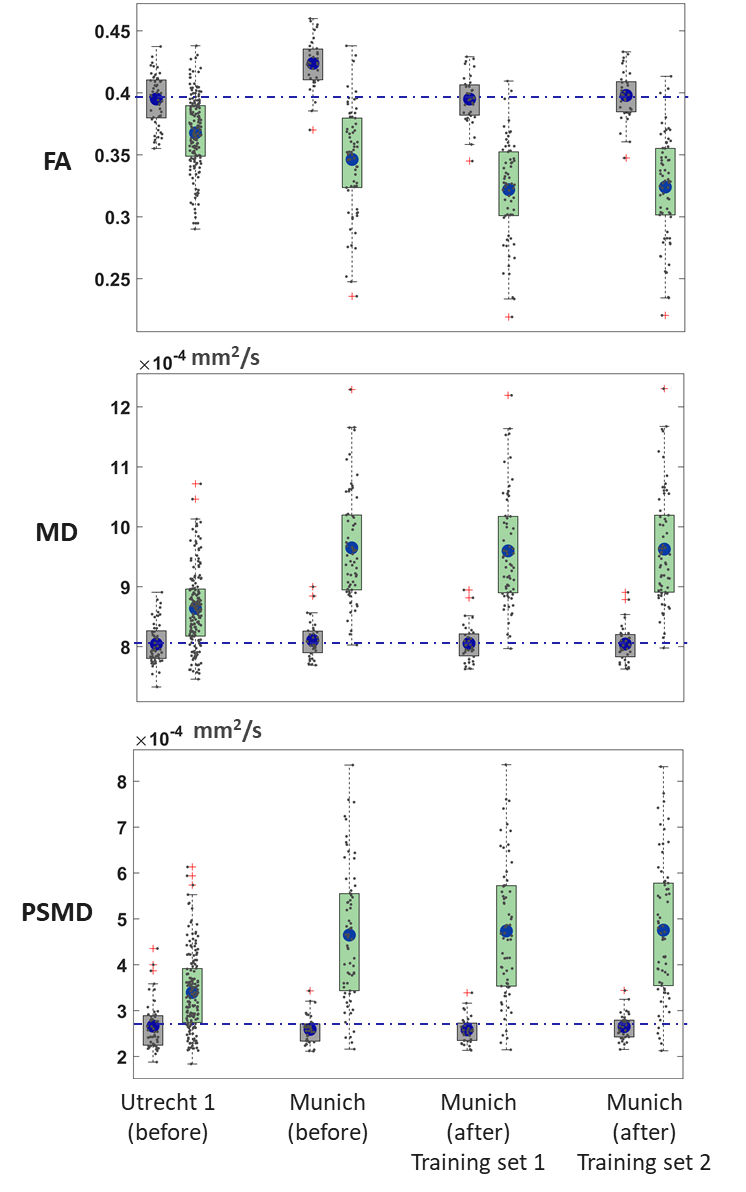
**

**Figure S3:** Average FA (top), MD (bottom) and PSMD of the white matter skeleton compared between patients (green) and controls (gray) within each site. Results are displayed for the reference site for the Munich site before and after harmonization using different training samples. Corresponding p-values and effect sizes are displayed in Table S4 The dashed line indicates the mean value of controls of the reference site and blue maker in each boxplot represent the mean of that group.

**Table S4:** Effect sizes between patients and controls for the Munich site, before and after harmonizing the data with different sets of training controls.

|  | | **Utrecht1** | **Hong Kong before** | **Hong Kong after** | **%**  **Change** | **Munich**  **before** | **Munich**  **After**  **(Training set 1)** | **%**  **Change** | **Munich**  **after**  **(Training set 2)** | **% Change** |
| --- | --- | --- | --- | --- | --- | --- | --- | --- | --- | --- |
| FA | P-value | 1.4×10^-11^ | 0.434 | 0.39 |  | 3.17×10^-21^ | 2.30×10^-21^ |  | 6×10^-21^ |  |
|  | Effect size (Cohen’s d) | -0.96 | -0.25 | -0.27 | 8 | -2.07 | -2.09 | 1 | -2.07 | 0 |
|  | CI | (-1.29, -.64) | (-.87, 0.37) | (-.88,0.35) |  | (-2.57, -1.57) | (-2.59, -1.59) |  | (-2.51, -1.52) |  |
| MD | P-value | 6.29×10^-10^ | 0.17 | 0.13 |  | 9.57×10^-21^ | 6.29×10^-21^ |  | 7.3×10^-21^ |  |
|  | Effect size (Cohen’s d) | 1.02 | 0.44 | 0.48 | 9 | 1.99 | 2 | 1 | 2 | 1 |
|  | CI | (.63, 1.34) | (-.19, 1.06) | (-.15, 1.11) |  | (1.49, 2.49) | (1.49, 2.48) |  | (1.49, 2.48) |  |
| PSMD | P-value | 3.146×10^-12^ | 0.059 | 0.036 |  | 3.601×10^-17^ | 5.971×10^-18^ |  | 5.6 ×10^-18^ |  |
|  | Effect size (Cohen’s d) | 0.93 | 0.62 | 0.69 | 11 | 1.71 | 1.78 | 4 | 1.77 | 4 |
|  | CI | (.62, 1.25) | (-.02, 1.25) | (.05, 1.32) |  | (1.23, 2.18) | (1.29, 2.25) |  | (1.29, 2.24) |  |

## Part 6 - Statistical harmonization using ComBat

We compared the performance of RISH harmonization with a recently proposed statistical harmonization tool, ComBat (Fortin et al., 2017). We performed 3 experiments using different sets of covariates in the model. We used age and sex as biological covariates for all experiments. Our aim was to understand whether scanner related differences in matched controls between sites are removed, while preserving effect sizes between patients and controls within each site. In the first experiment, patients and controls were harmonized in the same batch using an additional covariate for “group” (e.g., patients = 2 vs. controls = 1). In the second experiment, controls and patients were also harmonized in the same batch, using a group covariate with values between 1 and 3 for controls, SVD patients and CADASIL patients, respectively. The aim of this analysis was to understand whether separating the subjects per disease type would enhance the performance of harmonization. Finally, in the third experiment we harmonized patients and controls in separate batches.

### Experiment 1: using a group covariate of patient and controls.

Figure S2 and Table S5 show results of ComBat harmonization using disease type as covariate. After harmonization, one-way ANOVA shows that significant differences still exist between matched controls of all sites: FA (F (3,120) = 31, p < 0.001), MD (F (3,120) = 40, p< 0.001), and PSMD (F (3,120) = 44, p< 0.001). Note that the controls (gray boxplots) do not fall on the same line before or after harmonization, suggesting that scanner related differences are not accurately removed. Furthermore, effect sizes between patients and controls are not always preserved after harmonization, and changes in effect size up to 96% were observed.


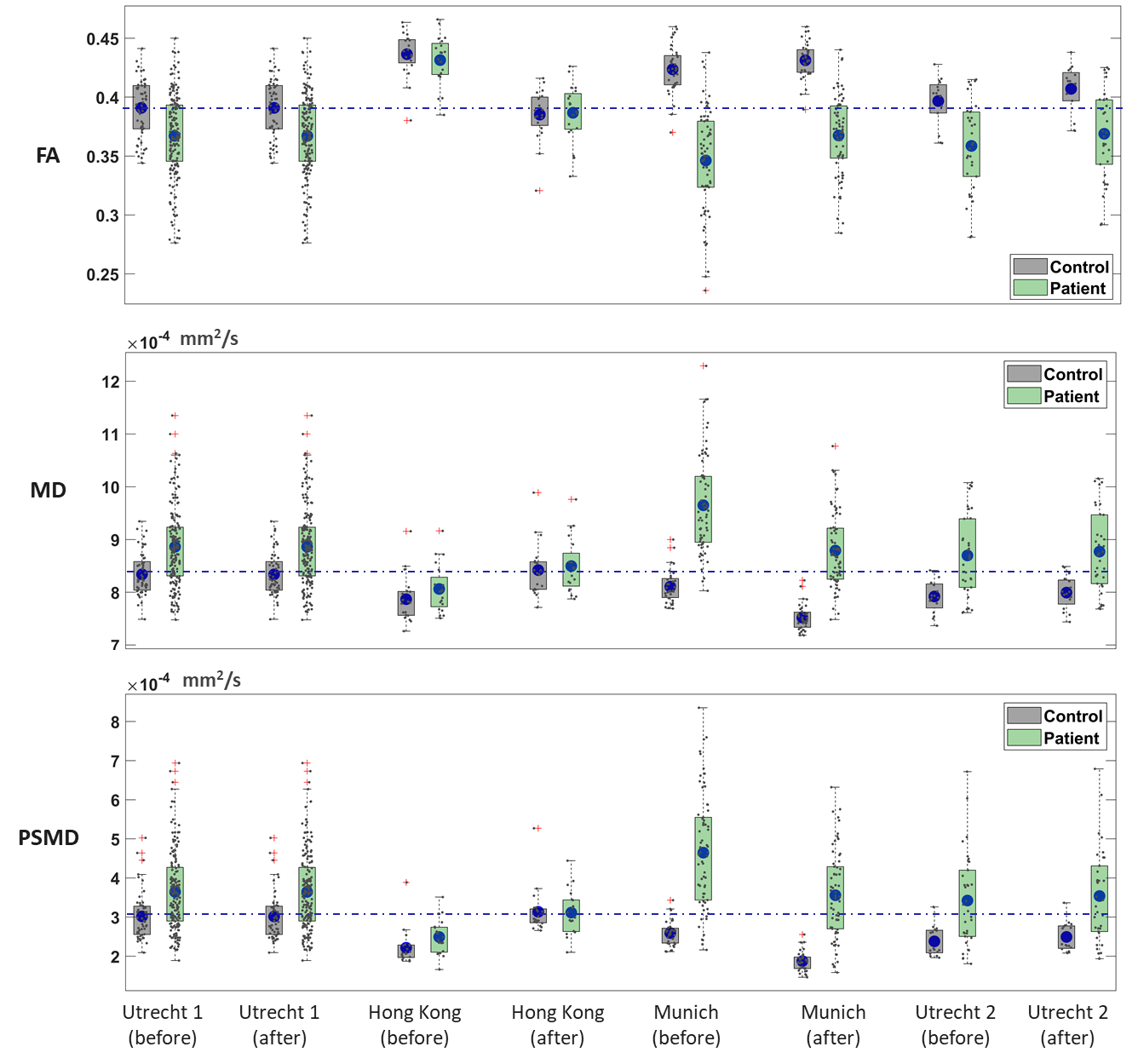


**Figure S4:** Harmonization using the ComBat method with age, sex and group (patients, controls) as covariates. Average FA (top), MD (bottom) and PSMD of the white matter skeleton compared between patients (green) and controls (gray) within each site, before and after harmonization. P-values and effect sizes are displayed in Table S4. The dashed line indicates the harmonized mean value of controls of the reference site and blue makers in each boxplot represents the mean of each group.

**Table S5:** Effect sizes between patients and controls within each site, before and after ComBat harmonization with age, sex and group (patients, controls) as covariates.

|  | | Utrecht1  before | Utrecht1  after | % Change | Hong Kong before | Hong Kong after | % Change | Munich  before | Munich  after | % Change | Utrecht2 before | Utrecht2 after | % Change |
| --- | --- | --- | --- | --- | --- | --- | --- | --- | --- | --- | --- | --- | --- |
| **FA** | P-value | 3.66×10^-9^ | 9.00×10^-6^ |  | .43 | .83 |  | 1.77×10^-16^ | 5.36×10^-18^ |  | 3.9×10^-4^ | 3.7×10^-4^ |  |
|  | Effect size (Cohen’s d) | -.97 | -.71 | 27 | -0.25 | .07 | 72 | -2.07 | -2.21 | -7 | -1.16 | -1.16 | 0 |
|  | CI (Cohen’s d) | (-1.29, -.64) | (-1.02, -.40) |  | (-.87, .37) | (-.55, .69) |  | (-2.57, -1.57) | (-2.73, -1.70) |  | (-1.79, -.52) | (-1.79, -.52) |  |
| **MD** | P-value | 6.30×10^-10^ | 2.00×10^-6^ |  | .17 | .63 |  | 1.14×10^-15^ | 5.16×10^-17^ |  | 2.80×10^-4^ | 2.9×10^-4^ |  |
|  | Effect size (Cohen’s d) | 1.01 | .76 | -25 | .44 | .15 | -66 | 1.99 | 2.12 | 7 | 1.19 | 1.18 | -1 |
|  | CI (Cohen’s d) | (.69, 1.34) | (.45, 1.08) |  | (-.19, 1.06) | (-.47, .77) |  | (1.50, 2.49) | (1.61, 2.62) |  | (.54, 1.82) | (.54, 1.82) |  |
| **PSMD** | P-value | 1.12×10^-8^ | 2.60×10^-5^ |  | .06 | .91 |  | 1.09×10^-12^ | 7.49×10^-14^ |  | .001 | 0.001 |  |
|  | Effect size (Cohen’s d) | .93 | .68 | -27 | 0.62 | -.04 | -94 | 1.71 | 1.82 | 6 | 1.03 | 1.65 | 60 |
|  | CI (Cohen’s d) | (.61, 1.25) | (.36, .99) |  | (-.02, .25) | (-.66, .58) |  | (1.23, 2.18) | (1.33, 2.30) |  | (.39, 1.65) | (.41, 1.67) |  |

### Experiment 2: using a group covariate of controls, SVD and CADASIL

Figure S3 and Table S6 show results of ComBat harmonization using disease type as covariate. Similar to the previous experiment, after harmonization, significant differences still persisted between matched controls of all sites: FA (F (3,120) = 7.8, p < 0.001), MD (F (3,120) = 9.4, p< 0.001), and PSMD (F (3,120) = 5.9, p< 0.001). Effect sizes between patients and controls were less affected by harmonization than in Experiment 1, showing that the inclusion of disease type as covariate can enhance the performance.


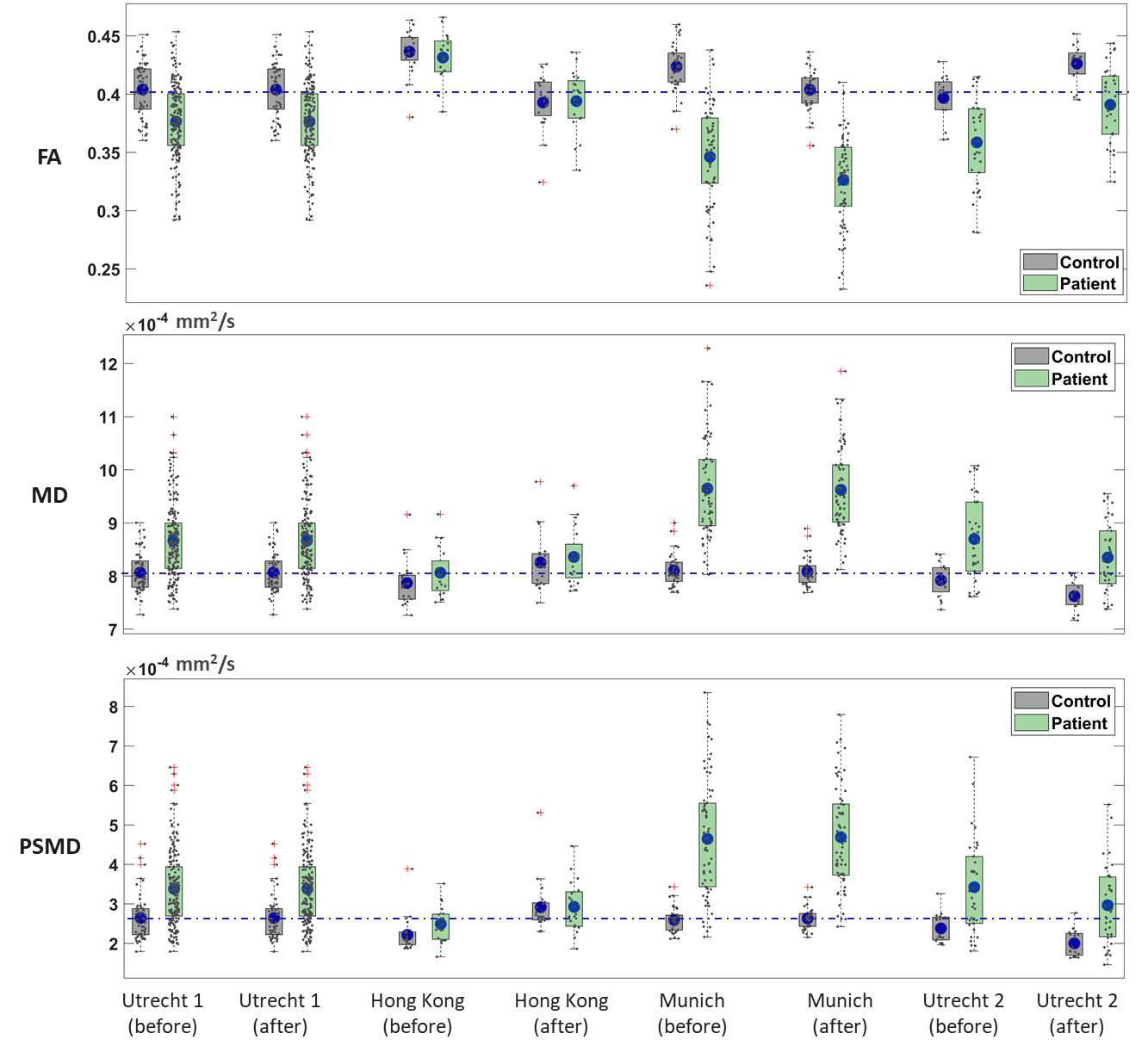


**Figure S5:** Harmonization using ComBat method with, age, sex and group (CADASIL, SVD, controls) as covariates. Average FA (top), MD (bottom) and PSMD of the white matter skeleton compared between patients (green) and controls (gray) within each site, before and after harmonization. P-values and effect sizes are displayed in Table S5. The dashed line indicates the harmonized mean value of controls of the reference site and blue makers in each boxplot represents the mean of each group.

**Table S6:** Effect sizes between patients and controls within each site, before and after ComBat harmonization with age, sex and group (CADASIL, SVD, controls) as covariates.

|  | | Utrecht1  before | Utrecht1  after | % Change | Hong Kong before | Hong Kong after | % Change | Munich  before | Munich  after | % Change | Utrecht2 before | Utrecht2 after | % Change |
| --- | --- | --- | --- | --- | --- | --- | --- | --- | --- | --- | --- | --- | --- |
| **FA** | P-value | 3.66×10^-9^ | 4.88×10^-8^ |  | 0.43 | 0.88 |  | 1.77×10^-16^ | 1.70×10^-19^ |  | 3.9×10^-4^ | 1.40×10^-4^ |  |
|  | Effect size (Cohen’s d) | -0.97 | -0.89 | 8 | -0.25 | 0.05 | 80 | -2.07 | -2.36 | -14 | -1.16 | -1.26 | -9 |
|  | CI (Cohen’s d) | (-1.29, -.64) | (-1.21, -.57) |  | (-.87, .37) | (-.57, .67) |  | (-2.57, -1.57) | (-2.88, -1.83) |  | (-1.79, -.52) | (-1.89, -.61) |  |
| **MD** | P-value | 6.30×10^-10^ | 1.01×10^-8^ |  | 0.17 | 0.54 |  | 1.14×10^-15^ | 1.38×10^-18^ |  | 2.80×10^-4^ | 1.02×10^-4^ |  |
|  | Effect size (Cohen’s d) | 1.01 | 0.94 | -7 | 0.44 | 0.19 | -57 | 1.99 | 2.27 | 14 | 1.19 | 1.28 | 8 |
|  | CI (Cohen’s d) | (.69, 1.34) | (.62, 1.26) |  | (-.19, 1.06) | (-.43, .82) |  | (1.50, 2.49) | (1.75, 2.79) |  | (.54, 1.82) | (.63, 1.93) |  |
| **PSMD** | P-value | 1.12×10^-8^ | 1.12×10^-8^ |  | 0.06 | 0.97 |  | 1.09×10^-12^ | 2.17×10^-15^ |  | 0.001 | 5.41×10^-4^ |  |
|  | Effect size (Cohen’s d) | 0.93 | 0.85 | -9 | 0.62 | 0.01 | -98 | 1.71 | 1.97 | 15 | 1.03 | 1.12 | 9 |
|  | CI (Cohen’s d) | (.61, 1.25) | (.36, .99) |  | (-.02, .25) | (-.66, .58) |  | (1.23, 2.18) | (1.33, 2.30) |  | (.39, 1.65) | (.41, 1.67) |  |

### Experiment 3: patients and controls harmonized in separate batches.

Figure S4 and Table S7 shows results of ComBat harmonization when patients and controls are harmonized separately. Contrary to the previous experiments, after harmonization, differences in FA, MD and PSMD between matched controls of all sites were removed (all p>0.05). Note that control groups (gray boxes) fall on the same line after harmonization. However, effect sizes between patients and controls were severely disrupted by harmonization. For example, for the Hong site where FA differences were not initially significant (Cohen’s = -0.25, p = .43), the effect size was inflated after harmonization (Cohen’s = -2.11, p = 6.74×10-8).


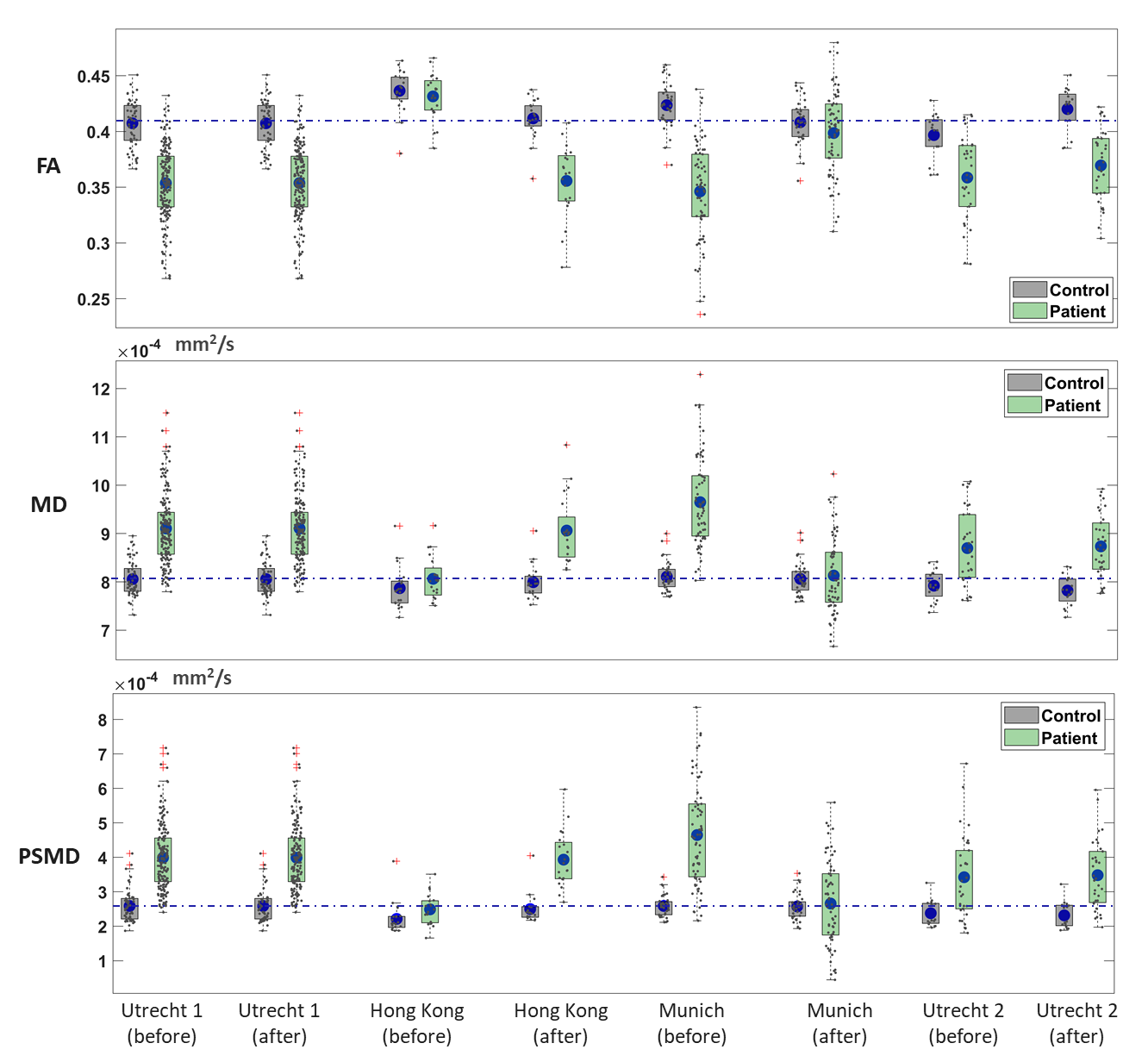


**Figure S6:** Harmonization using ComBat method, with patients and controls harmonized separately using age, sex and group as covariates. Average FA (top), MD (bottom) and PSMD of the white matter skeleton compared between patients (green) and controls (gray) within each site, before and after harmonization. P-values and effect sizes are displayed in Table S6. The dashed line indicates the harmonized mean value of controls of the reference site and blue makers in each boxplot represent the mean of each group.

**Table S7:** Effect sizes between patients and controls within each site, before and after ComBat harmonization with patients and controls harmonized separately. Age and sex were used as covariates.

|  | | Utrecht1  before | Utrecht1  after | % Change | Hong Kong before | Hong Kong after | % Change | Munich  before | Munich  after | % Change | Utrecht2 before | Utrecht2 after | % Change |
| --- | --- | --- | --- | --- | --- | --- | --- | --- | --- | --- | --- | --- | --- |
| **FA** | P-value | **3.66×10^-9^** | **1.32×10^-22^** |  | **0.43** | **6.74×10^-8^** |  | **1.77×10^-16^** | **0.15** |  | **3.9×10^-4^** | **3.85×10^-7^** |  |
|  | Effect size (Cohen’s d) | -0.97 | -1.72 | -77 | -0.25 | -2.11 | -744 | -2.07 | -0.3 | 86 | -1.16 | -1.78 | -53 |
|  | CI (Cohen’s d) | (-1.29, -.64) | (-2.07, -1.37) |  | (-.87, .37) | (-2.88, -1.32) |  | (-2.57, -1.57) | (-.71, .11) |  | (-1.79, -.52) | (-2.47, -1.08) |  |
| **MD** | P-value | 6.30×10^-10^ | 1.21×10^-20^ |  | 0.17 | 3.17×10^-7^ |  | 1.14×10^-15^ | 0.61 |  | 2.80×10^-4^ | 3.00×10^-6^ |  |
|  | Effect size (Cohen’s d) | 1.01 | 1.62 | 60 | 0.44 | 1.96 | 345 | 1.99 | 0.11 | -94 | 1.19 | 1.61 | 35 |
|  | CI (Cohen’s d) | (.69, 1.34) | (1.28, 1.96) |  | (-.19, 1.06) | (1.18, 2.71) |  | (1.50, 2.49) | (-.31, .52) |  | (.54, 1.82) | (.93, 2.28) |  |
| **PSMD** | P-value | 1.12×10^-8^ | 2.19×10^-20^ |  | 0.06 | 2.51×10^-8^ |  | 1.09×10^-12^ | 0.69 |  | 0.001 | 4.50×10^-5^ |  |
|  | Effect size (Cohen’s d) | 0.93 | 1.61 | 73 | 0.62 | 2.21 | 256 | 1.71 | 0.08 | -95 | 1.03 | 1.36 | 32 |
|  | CI (Cohen’s d) | (.61, 1.25) | (.36, .99) |  | (-.02, .25) | (-.66, .58) |  | (1.23, 2.18) | (1.33, 2.30) |  | (.39, 1.65) | (.41, 1.67) |  |

## Part 7 - Prevalence of WMH in training controls.


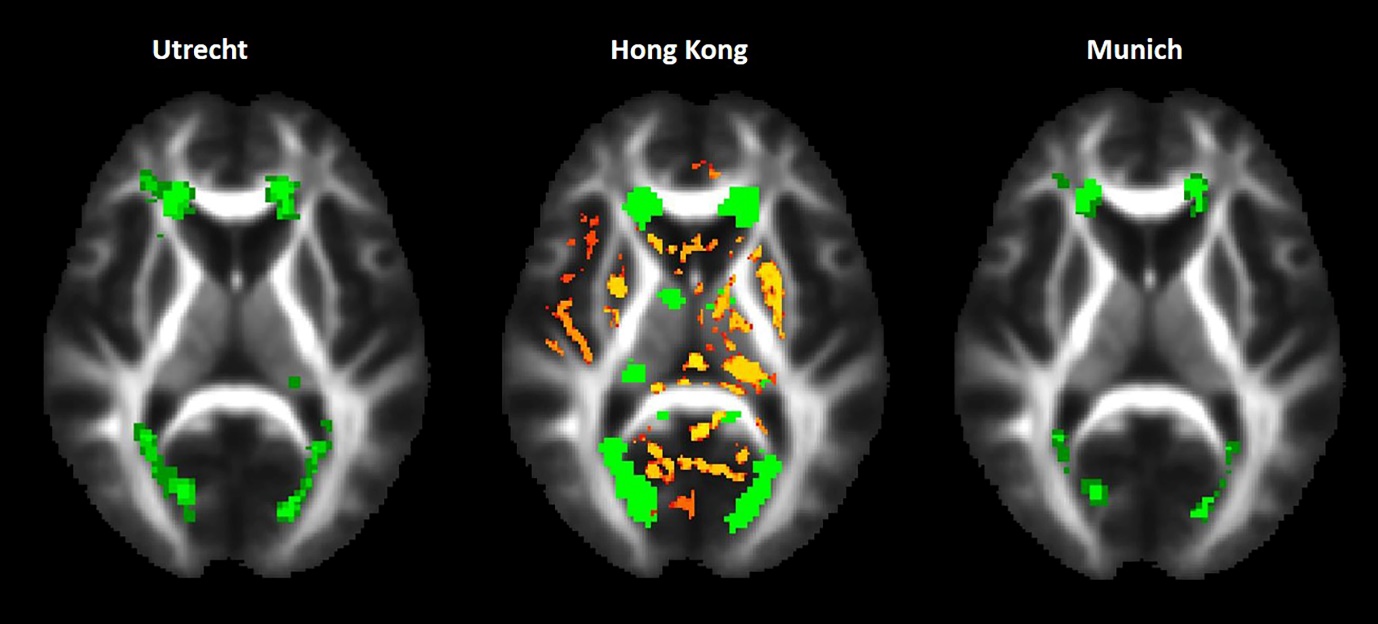


**Figure S7:** White matter hyperintensities (WMHs) observed in training controls of different sites. The green areas correspond to WMHs that occur in at least 25% of the subjects. For the Hong Kong site, the red-yellow colormap indicates areas where MD differences persisted after harmonization (p<0.05 when compared to the reference site).
